# A twenty-eight-day evaluation of cytotoxic drug vapor containment in drug-binding closed-system transfer device

**DOI:** 10.3389/fpubh.2025.1400571

**Published:** 2025-06-02

**Authors:** Dekel Navarro, Daniel Epstein

**Affiliations:** Nextar Chempharma Solutions Ltd., Ness Ziona, Israel

**Keywords:** activated carbon, closed system transfer device, drug binding technology, drug vapors, hazardous drugs

## Abstract

**Objective:**

Several studies have demonstrated that hazardous drugs can evaporate even at ambient temperature during their preparation in healthcare facilities, potentially posing a health risk for clinicians. The National Institute for Occupational Safety and Health (NIOSH) has defined closed system transfer device (CSTD) performance as preventing the release of hazardous drugs in the form of vapor, aerosol, or droplets. Most CSTDs can be used to store drugs for up to 7 days after their preparation. However, as some drugs are stable for more than 7 days, the CSTD usage period represents a limiting factor leading to residual drug waste. We investigated whether the Chemfort^®^ CSTD with the ToxiGuard^®^ system, an activated carbon matrix, minimizes the exposure to hazardous drug vapors or aerosols that may be released for 28 days after drug preparation.

**Methods:**

Cyclophosphamide, a cytotoxic drug with relatively high vapor pressure was chosen as the representative drug to demonstrate vapor escape prevention. Testing was performed using intact vial adaptors (with ToxiGuard^®^) after incubation for 28 days, intact vial adaptors (with ToxiGuard^®^) without incubation, a vial adaptor from which the carbon matrix was removed (positive control) and a vial adaptor containing only water (negative control). After each test, the components were rinsed or swabbed to test for cyclophosphamide contamination.

**Results:**

No escaped cyclophosphamide was detected in the tests performed using Chemfort^®^ with intact ToxiGuard^®^. In the system tested without ToxiGuard^®^, 110.3 ng of escaped cyclophosphamide were detected.

**Conclusion:**

The intact ToxiGuard^®^, as part of the Chemfort^®^ vial adaptor, prevents release of hazardous cyclophosphamide from the vial into the environment for up to 28 days. This result supports potential extension of its usage period and potential drug waste prevention with associated cost savings.

## Introduction

Cytotoxic drugs are characterized by their ability to disrupt the cell cycle and induce apoptosis in rapidly dividing cells (1). While this mechanism of action is vital for combating cancer, it also poses significant health hazards to individuals who come into contact with these substances. Healthcare personnel, including pharmacists, nurses, and pharmacy technicians, are routinely exposed to cytotoxic drugs during their daily clinical duties (2–6). This exposure can occur at various stages of drug handling, such as compounding, administration, and waste disposal (7).

The consequences of inadvertent exposure to cytotoxic agents can be dire. These substances are associated with a range of adverse health effects, including acute and chronic toxicities, carcinogenicity, mutagenicity, and reproductive toxicity (collectively called CMR hazards) (5, 8, 9). Healthcare personnel exposed to cytotoxic drugs face an elevated risk of developing a spectrum of health problems, ranging from local effects such as skin or eye irritation, headache, nausea or dizziness, and acute toxicity to long-term health complications and morbidity such as cancer (10–13).

Recognizing the significance of this occupational health concern, healthcare institutions, regulatory agencies, and healthcare workers themselves have increasingly sought ways to mitigate the risks associated with cytotoxic drug handling (14). Strategies for minimizing exposure range from the use of personal protective equipment, stringent handling protocols, and staff training initiatives to closed system transfer devices (CSTDs) (15–20). The National Institute for Occupational Safety and Health (NIOSH) 2004 alert (21) and the 2008 revision of Chapter <797> of the United States Pharmacopeia (USP) “Pharmaceutical Compounding—Sterile Preparations” (22) recommended the use of CSTD together with other safe-handling guidelines. The European Commission’s Guidelines for Good Manufacturing Practice for Medicinal Products for Human and Veterinary Use (23), also recommends the usage of closed systems depending on risk assessment results.

CSTDs employ a mechanical barrier to facilitate the process of reconstituting drug powder and transferring a drug solution into empty or pre-filled containers, such as infusion bags, bottles or syringes. The dual objectives of CSTDs are to preserve product sterility and to safeguard healthcare professionals from potential exposure to CMR toxic substances (20, 21, 24). USP <800> “Hazardous Drugs - Handling in Healthcare Settings” (22) supports previous compounding recommendations outlined in USP <797> and mandates CSTD use for hazardous drug administration when the dosage form enables using such devices (20).

Chemfort® (Simplivia Healthcare Ltd., Kiryat Shmona, Israel; branded OnGuard® 2 in the USA) is a CSTD with a unique ToxiGuard® technology. It comprises two components: (1) a hydrophobic acrylic copolymer membrane with a pore size of 0.2 microns (Versapor®) that maintains the sterility of the drug in the vial during pressure equalization (25, 26), protects the drug from introduction of particulate matter, and prevents release of liquids and aerosols; and (2) an activated carbon layer (Flexzorb™) that mechanically prevents the release of drug vapor during preparation and administration. The currently approved usage period of the Chemfort® CSTD components is 7 days.

Cyclophosphamide is frequently prepared in hospitals and has a relatively high vapor pressure of 4.4 × 10^−5^ mmHg compared to other hazardous drugs (27). Therefore, it can serve as an appropriate example for testing scenarios of escaped drug vapors during cytotoxic drug preparations. A previous study has shown that the Chemfort® vial adaptor prevents the escape of cyclophosphamide drug vapor even after 3 years of simulated aging of the vial adaptors and 7 days of exposure to drug vapors (26), in line with its approved shelf life and usage period. In that study, 5 positive control vial adaptors lacking activated carbon layers were compared to 5 freshly manufactured vial adaptors and 2 vial adaptors at the end of their shelf life all connected to cyclophosphamide vials reconstituted immediately before testing, and 5 vial adaptors at the end of their shelf life connected to cyclophosphamide vials reconstituted 7 days prior to testing. Cyclophosphamide release was detected only for the samples lacking activated carbon, at quantities ranging from 22 ng to 112 ng.

Several drug stability studies have shown that reconstituted cyclophosphamide remains stable for long periods of time. For example, the decomposition of cyclophosphamide stored at 2–8°C was <1 and <1.11% after 7 days and 14 days, respectively (28). Cyclophosphamide dissolved in dimethyl sulfoxide and stored at 4°C remained 100% stable even after 3 months (29). Accordingly, some multidose vials of cyclophosphamide are approved for storage of up to 28 days following first use (30). Furthermore, a recent study has shown the ability of Chemfort® vial adaptors to maintain drug vial sterility for up to 28 days after first puncture (31) Therefore, a key question is whether the performance of the Chemfort® vial adaptors can be extended beyond 7 days of exposure to drug vapors.

In this study we examined whether Chemfort® with the ToxiGuard® system can minimize exposure to hazardous drug vapors or aerosols that may be released or generated during drug preparation with the system and for the subsequent 28 days. The performance of devices at the end of their 3-year shelf life was also examined.

## Materials and methods

A model system was developed to establish extreme laboratory conditions (26), in which drug vapors are generated to a much larger extent than in typical working environments in hospitals and pharmacies. These extreme conditions included heating the vials in a closed test chamber to 50° C and introducing a constant flow of nitrogen into the vials to enhance the generation of drug aerosols and vapors. Any potential drug vapors present in the air released from the Chemfort® vial adaptors were collected and then analyzed by liquid chromatography with tandem mass spectrometry (LC/MS/MS).

Before the study began, all product vials were cleaned and dried to remove any residues from production or shipping by rinsing with 5% Alconox detergent solution (Sigma-Aldrich, St. Louis, MO, USA) followed by wiping with 70% isopropyl alcohol wipes (PROSAT® Sterile™, Contec Inc. Spartanburg, SC, USA). The bottles were then air dried before proceeding with the vapor trapping experiment.

Four different setups were used to examine whether drug vapors escape the system during drug preparation: (1) cyclophosphamide vials equipped with intact vial adaptors incubated in an oven at 30° C for 28 days after reconstitution (5 samples), (2) cyclophosphamide vials equipped with intact vial adaptors that were tested immediately after reconstitution (2 samples), (3) cyclophosphamide vial equipped with a vial adaptor lacking the carbon layer of the ToxiGuard® that was tested immediately after reconstitution (positive control, 1 sample), and (4) a vial containing water equipped with an intact vial adaptor (1 replicate) incubated in an oven at 30° C for 28 days (negative control, 1 sample). Sample sizes for setups 2 and 3 were smaller than for 1, as these setups were tested using the same equipment in a previous publication (26). Setups 2 and 3 were included in this study to validate the experimental process, but resources were invested in a larger sample size for the novel setup. All vial adaptors tested had previously undergone simulated aging equivalent to 3 years. This was achieved by incubating the vial adaptors at 55° C for 135 days according to the Standard Guide for Accelerated Aging of Sterile Medical Device Packages (32). Three years is the approved shelf life of the tested Chemfort® devices.

Cyclophosphamide (Endoxan 500 mg, Baxter, Deerfield, IL, USA) was used as a representative drug to test the ability of Chemfort® to minimize exposure to hazardous drug vapors or aerosols released or generated during drug compounding and vial storage.

### Vapor trapping

**Figure 1:**
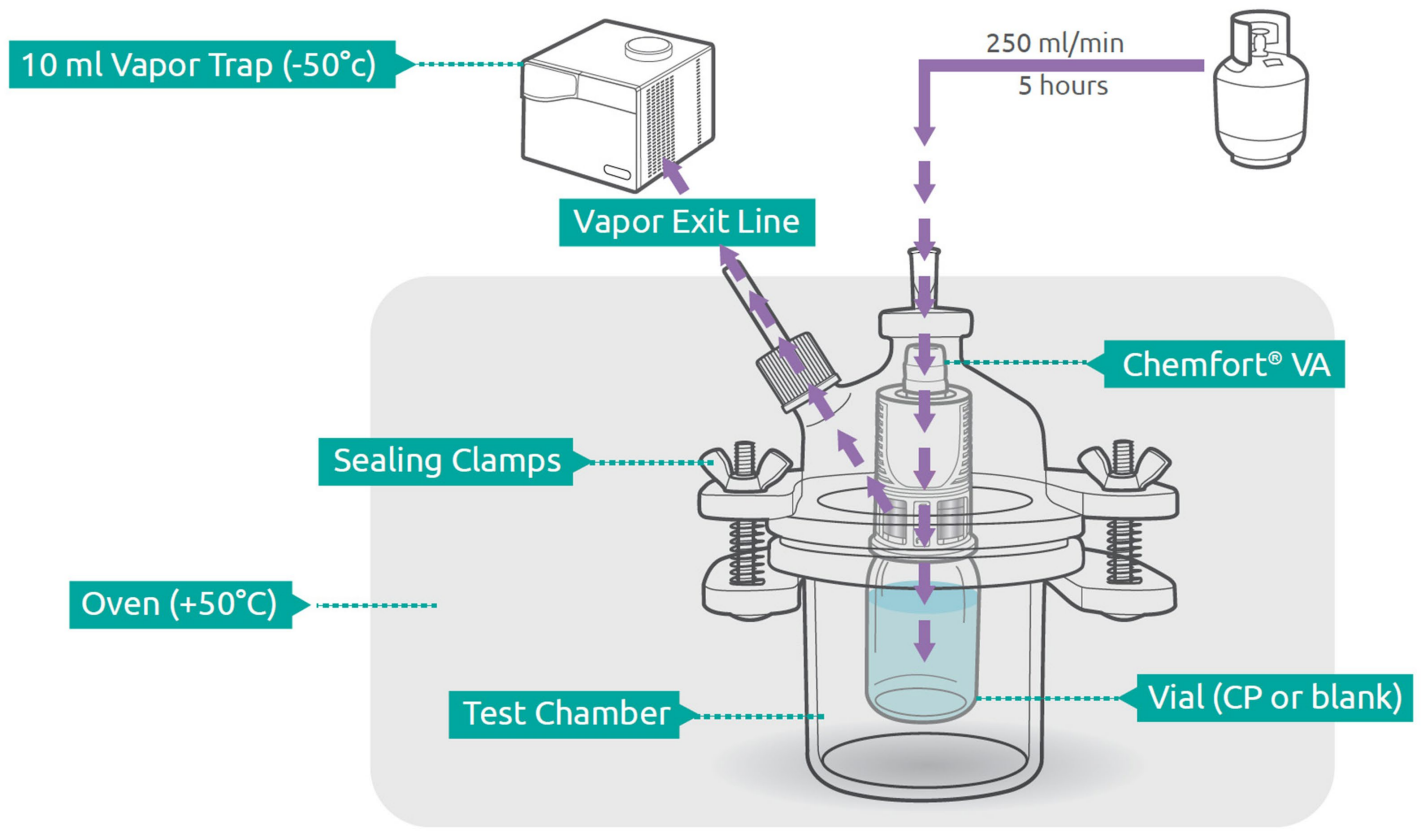
Schematic of experimental setup. Components are out of scale. Adapted from Levin and Sessink (26) with permission. VA, vial adaptor; CP, cyclophophamide.

To measure the amount of vapor that escapes the Chemfort® system during cyclophosphamide preparation, a vapor trapping system was set up. The vial equipped with a Chemfort® vial adaptor was placed in a reactor vessel. A 0.80⨯38-mm 21G needle was introduced through both the reactor vessel stopper and the vial adaptor septum such that an external stream of nitrogen gas (250 mL/min) flowed into the liquid pathway of the vial adaptor (Figure 1). In this manner, the nitrogen gas entered the vial via the liquid pathway of the vial adaptor, forcing air to exit the vial through the air pathway of the vial adaptor into the reactor vessel. Air exiting the reactor vessel passed through a gas outlet line and tubing (PHARMA-50 Tubing, ID 4.76 mm, OD 9.53 mm, Dow Corporate, Midland, MI, USA) into a cold trap collection vessel (Labconco, Kansas City, MO, USA). The reactor vessel was kept in an oven at 50° C and the collection trap was immersed in a cooling bath kept at approximately −50° C using a chiller. Analyte vapors were collected from the nitrogen stream for 5 h.

### Collection and recovery of escaped cyclophosphamide vapor

To collect the escaped drug for quantification, at the end of the collection period all potentially contaminated surfaces were wiped (vial, vial adaptor, and reactor vessel) or rinsed (tubing and collection vessel) as follows. The round-bottom collection flask together with the attached connector were rinsed with 10 mL diluent, and the tubing along with the tube connector attached to the reaction vessel were rinsed with 10 mL diluent. Each portion of diluent was collected and analyzed separately. Swabs (Low Total Organic Carbon Alpha® Polyester Knit TX714K, Texwipe, Kernersville, NC, USA) were soaked in diluent (1:1 methanol:water), then the interior surfaces of the reactor vessel top (swab 1) and bottom (swab 2), and drug vial exterior together with vial adaptor exterior (swab 3) were wiped with separate swabs. Next, each swab was placed separately in a tube, extracted with 10 mL of diluent (1:1 methanol:water), and sonicated for ten minutes.

The samples were removed and stored in a refrigerator at 2–8° C for either 2 or 5 days until they were analyzed.

### Liquid chromatography with tandem mass spectrometry analysis

Following extraction, 1,000 μL of the solution were mixed in a high-pressure liquid chromatography (HPLC) vial with 50 μL of internal standard solution (0.5 μg/mL D4-cyclophosphamide, Toronto Research Chemicals, Toronto, ON, Canada).

Analysis was performed with a 3200 Q TRAP Linear Ion Trap Quadrupole MS/MS (Sciex, Framingham MA, USA) combined with an Agilent 1100 HPLC system (Agilent Technologies, Santa Clara, CA, USA).

A Phenomenex Synergi Polar-RP 80A, 4 *μ*, 2⨯100-mm separation column operated at 35°C was used with a flow of 0.65 mL/min. Elution was performed using a gradient of 10 mM ammonium formate and acetonitrile (Table 1). The total runtime was 7.5 min. The retention time was 2.8 min.

**Table 1:** Liquid chromatography gradient for cyclophosphamide elution.

Time (min)	10 mM ammonium formate solution (%)	Acetonitrile solution (%)
0	90	10
0.5	90	10
1	10	90
3	10	90
3.1	90	10
7.5	90	10

The QTRAP 3200 mass spectrometer was operated in MRM mode using electrospray ionization in positive ion mode (ESI). Desolvation temperature was 650°C with an ion spray voltage of 5,000 V and a nitrogen curtain gas flow of 10 L/min. Source gas flow was set at 30 L/min (nitrogen). The detector energies were set at declustering potential of 50 V and collision potential of 30 V using nitrogen as collision gas. Selected ions were 261.1 (parent) and 139.8 (fragment) for cyclophosphamide and 265.1 (parent) and 142.0 (fragment) for the internal standard d4-cyclophosphamide. The limit of detection for cyclophosphamide was <0.2 ng on swabs and 0.02 ng/mL in solution.

## Results

Before vapor trapping, no cyclophosphamide was found in wash solutions or swab extract solutions of any of the experimental setups. The results of cyclophosphamide detection in vapor traps after drug preparation and incubation are shown in Table 2. As expected, no cyclophosphamide was detected in the test with the negative control vial, which contained water only, and which was performed with an intact ToxiGuard®. No escaped cyclophosphamide was detected in the tests performed using Chemfort® with an intact ToxiGuard® - either immediately or after incubation for 28 days. In contrast, without the carbon layer of the ToxiGuard® component (tested immediately), a total of 110.3 ng of escaped cyclophosphamide were detected. Thus, an intact ToxiGuard®, as an integral part of the Chemfort® vial adaptor, prevents release of hazardous cyclophosphamide from the vial into the environment.

**Table 2:** Detection of cyclophosphamide (ng) on surfaces in the vapor trap system after 5 h of vapor trapping.

**Experimental setup**	**CSTD**	**Chemfort® with intact vial adaptors**	**Chemfort® with intact vial adaptors**	**Chemfort® with intact vial adaptor**	**Chemfort® with intact vial adaptor**
	**Number of replicates**	***N* = 5**	***N* = 2**	***N* = 1**	***N* = 1**
	**Barriers**	**ToxiGuard®**	**ToxiGuard®**	**No carbon matrix**	**ToxiGuard®**
	**Drug tested**	**Cyclophosphamide**	**Cyclophosphamide**	**Cyclophosphamide**	**Water**
	**Incubation period**	**28 days**	**0 days**	**0 days**	**28 days**
**Cyclophosphamide (ng) found in wash solutions and swab extract solutions of vapor trap components after vapor trapping**	**Round-bottom flask**	ND	ND	ND	ND
	**Vial adaptor and drug vial**	ND	ND	109.30	ND
	**Vessel top and connector**	ND	ND	0.29	ND
	**Vessel bottom**	ND	ND	ND	ND
	**Tubing and connector**	ND	ND	0.69	ND
	**Total**	ND	ND	110.30	ND

CSTD, closed system transfer device; ND, not detected. The limit of detection for cyclophosphamide was <0.2 ng.

## Discussion

Several studies have demonstrated that hazardous drugs can evaporate at ambient temperature during their preparation in healthcare facilities (3, 25, 33, 34). NIOSH defines CSTD performance by its ability to fully contain hazardous drugs in the form of vapor, aerosol, or droplets (21). The International Society of Oncology Pharmacy Practitioners (ISOPP) has also referred to prevention of the escape of hazardous drug or vapor concentrations outside the system in their definition of CSTDs (19). The use of a vapor containment performance protocol for CSTDs was also mentioned in European Parliament Policy Recommendations (35).

**Figure 2:**
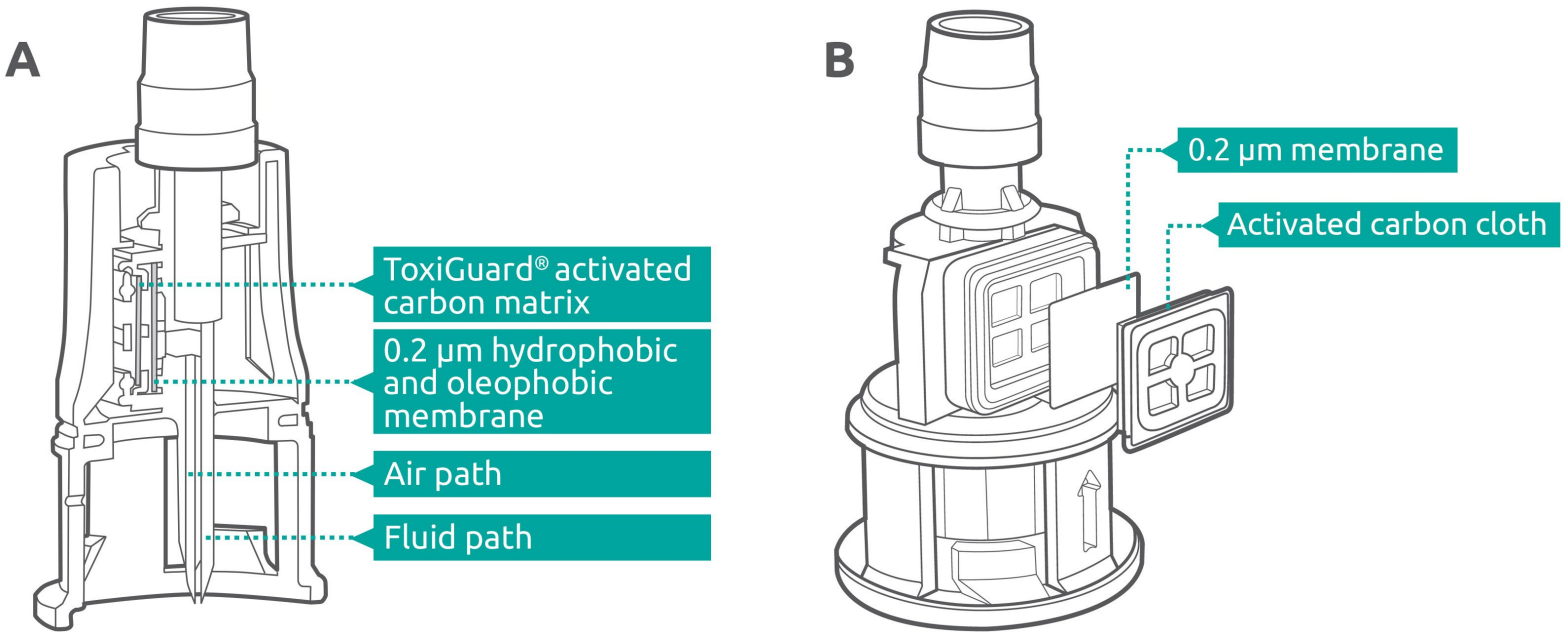
**(A)** Cross-section of the Chemfort® vial adaptor. **(B)** ToxiGuard® component of the Chemfort® vial adaptor.

The Chemfort® vial adaptor contains two channels; one of them serves as the air pathway for pressure equalization and the other as the liquid pathway for fluid transfer into and out of the vial (Figure 2A). The ToxiGuard® component of the vial adaptor is situated at the most exterior point of the air pathway and comprises a 0.2-micron hydrophobic membrane and a 100% activated carbon drug binding matrix (Zorflex activated carbon cloth), such that air exiting the vial passes first through the membrane and then through the carbon matrix (Figure 2B). The hydrophobic membrane blocks the passage of aqueous liquids and aerosols out of the air channel, while maintaining high air permeability. The manufacturing process for the activated carbon matrix results in a woven carbon cloth with a highly microporous structure and strong electrostatic forces. This matrix is highly efficient in adsorbing both organic and inorganic molecules from vapor that may pass through the 0.2-micron membrane, preventing their release into the environment.

The vapor containment ability of a ToxiGuard®-based CSTD (Chemfort®’s predecessor, Tevadaptor/OnGuard® in the USA) was demonstrated in accordance with the 2016 unified draft NIOSH protocol (36) using 2-phenoxyethanol as a surrogate for hazardous drugs. This compound represents a worse case model for hazardous drugs in terms of its vapor pressure, Henry’s volatility constant, chemical structure, and physicochemical behavior (27). The 2016 draft NIOSH protocol enables quantitative assessment of all types of CSTDs regardless of their technology (36). When using Tevadaptor, no vapor release was detected above the limit of quantification for the assay (<0.88 parts per billion) (27). In the current study, cyclophosphamide, a cytotoxic drug with relatively high vapor pressure was used to demonstrate vapor escape prevention.

According to the Chemfort® instructions for use, the vial adaptors may be used for up to 7 days following attachment to a drug vial. During this period, they are constantly exposed to drug vapor. In a prior study, the functionality of the carbon matrix was tested immediately after vial adaptors were attached to the vials for the preparation of cyclophosphamide and 5-fluorouracil and 7 days later for cyclophosphamide alone. Additionally, some of the vial adaptors were at the end of their 3-year shelf life. The results demonstrated that one week of exposure to hazardous drug vapor did not reduce the adsorption capability of the activated carbon matrix, as no release of cyclophosphamide was detected, even after 7 days exposure at the end of the vial adaptor shelf life (26). In the previous study, between 22 and 112 ng of cyclophosphamide were detected for positive control samples. The quantity determined for the positive control in this study (110 ng) was within the same range. The variability among results in the previous study indicates that without the activated carbon layer of ToxiGuard® contamination is consistently detected at the extreme testing conditions applied. The level of contamination is, however, unpredictable and highly variable. The current study has shown that the activated carbon layer of the Chemfort® vial adaptor ToxiGuard® plays an important role in preventing drug vapor escape of one of the most volatile hazardous drugs under extreme conditions, even after 28 days of incubation at the end of the vial adaptors’ shelf life. The test conditions to release the drug vapors were evaluated by collecting vapors and aerosols using a vial adaptor lacking an activated carbon layer (without incubation). Under this test condition cyclophosphamide was released and spread outside the vials, contaminating the vial adaptor and drug vial, the vessel top and its connector, and the tubing and its connector. Interestingly, contamination was not detected in the round bottom flask or vessel bottom. However, investigation of the spread of contamination to specific locations is beyond the scope of this study. Given the extreme conditions, the relative volatility of the tested drug, and the age of the devices tested, these results can represent a worst-case usage of the Chemfort® vial adaptor, suggesting that vapors of all other hazardous drugs would most likely be fully contained under clinical use conditions when using Chemfort® up to 3 years after its production and for a duration of up to 28 days.

It is important to note that the findings of this study apply only to the Chemfort® CSTD. Additional studies to evaluate whether other CSTDs have the same capabilities are recommended.

## Conclusion

The findings of the study indicate that the Chemfort® CSTD prevents the escape of vapors of hazardous drug preparations for up to 28 days, potentially helping to protect healthcare personnel handling of such drugs, even when stored for an extended period of time.

## Data Availability

The raw data supporting the conclusions of this article will be made available by the authors, without undue reservation.
